# Supplementary material for: Modeling glioblastoma heterogeneity as a dynamic network of cell states
Source: Mol Syst Biol. 2021 Sep 16;17(9):e10105. doi: 10.15252/msb.202010105 (PMC8444284; doi:10.15252/msb.202010105)
Supplement: Supplementary file 6 — Source Data for Figure 5 [file MSB-17-e10105-s004.zip › Figure5A_sourcedata/GSEA_3071/nosigdb_stateI.GseaPreranked.1622795145664/RICHARDS_ZHONG_NATURE_2018_MICROGLIA_UPREG.html]

Details for gene set RICHARDS\_ZHONG\_NATURE\_2018\_MICROGLIA\_UPREG[GSEA]

|  || Dataset | state13071 |
| Phenotype | NoPhenotypeAvailable |
| Upregulated in class | na\_pos |
| GeneSet | RICHARDS\_ZHONG\_NATURE\_2018\_MICROGLIA\_UPREG |
| Enrichment Score (ES) | 0.35657883 |
| Normalized Enrichment Score (NES) | 2.2179265 |
| Nominal p-value | 0.0 |
| FDR q-value | 4.2332447E-4 |
| FWER p-Value | 0.009 |
Table: GSEA Results Summary

  

Fig 1: Enrichment plot: RICHARDS\_ZHONG\_NATURE\_2018\_MICROGLIA\_UPREG      
 Profile of the Running ES Score & Positions of GeneSet Members on the Rank Ordered List

  

| PROBE | GENE SYMBOL | GENE\_TITLE | RANK IN GENE LIST | RANK METRIC SCORE | RUNNING ES | CORE ENRICHMENT || 1 | SPP1 |  |  | 0 | 2.808 | 0.0376 | Yes |
| 2 | SLC29A1 |  |  | 50 | 1.357 | 0.0412 | Yes |
| 3 | APOE |  |  | 60 | 1.294 | 0.0559 | Yes |
| 4 | PLXDC2 |  |  | 61 | 1.265 | 0.0728 | Yes |
| 5 | BHLHE41 |  |  | 69 | 1.218 | 0.0871 | Yes |
| 6 | SERPINF1 |  |  | 71 | 1.211 | 0.1030 | Yes |
| 7 | ZFP36L1 |  |  | 97 | 1.096 | 0.1102 | Yes |
| 8 | NEAT1 |  |  | 123 | 0.982 | 0.1160 | Yes |
| 9 | OLFML3 |  |  | 125 | 0.971 | 0.1287 | Yes |
| 10 | BIN1 |  |  | 127 | 0.967 | 0.1413 | Yes |
| 11 | CTSB |  |  | 129 | 0.964 | 0.1539 | Yes |
| 12 | NPC2 |  |  | 139 | 0.943 | 0.1639 | Yes |
| 13 | NABP1 |  |  | 154 | 0.907 | 0.1719 | Yes |
| 14 | ANXA4 |  |  | 190 | 0.847 | 0.1729 | Yes |
| 15 | XBP1 |  |  | 215 | 0.797 | 0.1764 | Yes |
| 16 | MAF |  |  | 227 | 0.773 | 0.1835 | Yes |
| 17 | B2M |  |  | 241 | 0.744 | 0.1896 | Yes |
| 18 | GSN |  |  | 265 | 0.720 | 0.1925 | Yes |
| 19 | S100A11 |  |  | 275 | 0.707 | 0.1993 | Yes |
| 20 | DAB2 |  |  | 277 | 0.702 | 0.2084 | Yes |
| 21 | RBMS1 |  |  | 285 | 0.697 | 0.2156 | Yes |
| 22 | TAPBP |  |  | 287 | 0.692 | 0.2246 | Yes |
| 23 | GRN |  |  | 321 | 0.655 | 0.2236 | Yes |
| 24 | LAMP2 |  |  | 352 | 0.629 | 0.2231 | Yes |
| 25 | PLTP |  |  | 374 | 0.611 | 0.2251 | Yes |
| 26 | RASSF4 |  |  | 384 | 0.603 | 0.2305 | Yes |
| 27 | ANXA5 |  |  | 392 | 0.597 | 0.2364 | Yes |
| 28 | IFNGR1 |  |  | 407 | 0.580 | 0.2400 | Yes |
| 29 | KLF2 |  |  | 428 | 0.571 | 0.2417 | Yes |
| 30 | HERPUD1 |  |  | 436 | 0.561 | 0.2472 | Yes |
| 31 | ELF1 |  |  | 439 | 0.558 | 0.2541 | Yes |
| 32 | CTSL |  |  | 440 | 0.558 | 0.2615 | Yes |
| 33 | LY96 |  |  | 451 | 0.547 | 0.2659 | Yes |
| 34 | JUNB |  |  | 454 | 0.546 | 0.2726 | Yes |
| 35 | SP100 |  |  | 466 | 0.538 | 0.2766 | Yes |
| 36 | CTSD |  |  | 474 | 0.531 | 0.2816 | Yes |
| 37 | LIMS1 |  |  | 498 | 0.514 | 0.2816 | Yes |
| 38 | RIN2 |  |  | 499 | 0.513 | 0.2885 | Yes |
| 39 | CTSS |  |  | 509 | 0.502 | 0.2926 | Yes |
| 40 | TSC22D3 |  |  | 513 | 0.500 | 0.2984 | Yes |
| 41 | BLVRB |  |  | 535 | 0.488 | 0.2987 | Yes |
| 42 | VMP1 |  |  | 545 | 0.483 | 0.3025 | Yes |
| 43 | HTRA1 |  |  | 555 | 0.477 | 0.3062 | Yes |
| 44 | FTL |  |  | 564 | 0.473 | 0.3102 | Yes |
| 45 | HSPA1A |  |  | 566 | 0.473 | 0.3162 | Yes |
| 46 | RCN3 |  |  | 579 | 0.466 | 0.3189 | Yes |
| 47 | RHOB |  |  | 609 | 0.447 | 0.3163 | Yes |
| 48 | CD63 |  |  | 618 | 0.444 | 0.3198 | Yes |
| 49 | KLF6 |  |  | 647 | 0.433 | 0.3173 | Yes |
| 50 | EGR1 |  |  | 652 | 0.432 | 0.3219 | Yes |
| 51 | TPP1 |  |  | 661 | 0.429 | 0.3253 | Yes |
| 52 | BST2 |  |  | 677 | 0.421 | 0.3265 | Yes |
| 53 | FMNL3 |  |  | 685 | 0.419 | 0.3301 | Yes |
| 54 | LIPA |  |  | 700 | 0.412 | 0.3314 | Yes |
| 55 | MYH9 |  |  | 701 | 0.411 | 0.3369 | Yes |
| 56 | TLN1 |  |  | 731 | 0.402 | 0.3337 | Yes |
| 57 | ARPC1B |  |  | 743 | 0.400 | 0.3358 | Yes |
| 58 | LYST |  |  | 752 | 0.397 | 0.3387 | Yes |
| 59 | DUSP1 |  |  | 787 | 0.386 | 0.3339 | Yes |
| 60 | ATP6V0E1 |  |  | 788 | 0.386 | 0.3390 | Yes |
| 61 | JAG1 |  |  | 796 | 0.383 | 0.3421 | Yes |
| 62 | OSTF1 |  |  | 808 | 0.378 | 0.3439 | Yes |
| 63 | POLD4 |  |  | 810 | 0.378 | 0.3487 | Yes |
| 64 | FOS |  |  | 832 | 0.370 | 0.3474 | Yes |
| 65 | IFITM2 |  |  | 836 | 0.370 | 0.3514 | Yes |
| 66 | FOSB |  |  | 854 | 0.364 | 0.3513 | Yes |
| 67 | NEU1 |  |  | 897 | 0.351 | 0.3435 | Yes |
| 68 | TPT1 |  |  | 971 | 0.334 | 0.3264 | Yes |
| 69 | PLD3 |  |  | 980 | 0.331 | 0.3285 | Yes |
| 70 | CASP1 |  |  | 981 | 0.331 | 0.3329 | Yes |
| 71 | C3AR1 |  |  | 984 | 0.330 | 0.3367 | Yes |
| 72 | ZFHX3 |  |  | 1001 | 0.327 | 0.3364 | Yes |
| 73 | TAGAP |  |  | 1025 | 0.323 | 0.3339 | Yes |
| 74 | RNF213 |  |  | 1027 | 0.322 | 0.3379 | Yes |
| 75 | LGMN |  |  | 1031 | 0.321 | 0.3413 | Yes |
| 76 | LPIN2 |  |  | 1035 | 0.321 | 0.3447 | Yes |
| 77 | MYL12A |  |  | 1053 | 0.316 | 0.3439 | Yes |
| 78 | CD81 |  |  | 1059 | 0.315 | 0.3467 | Yes |
| 79 | IFI16 |  |  | 1062 | 0.315 | 0.3503 | Yes |
| 80 | CTSA |  |  | 1065 | 0.314 | 0.3539 | Yes |
| 81 | PPT1 |  |  | 1071 | 0.312 | 0.3566 | Yes |
| 82 | MGST2 |  |  | 1097 | 0.307 | 0.3533 | No |
| 83 | GSTK1 |  |  | 1113 | 0.304 | 0.3529 | No |
| 84 | CD164 |  |  | 1133 | 0.301 | 0.3513 | No |
| 85 | NR3C1 |  |  | 1162 | 0.297 | 0.3470 | No |
| 86 | ATM |  |  | 1181 | 0.292 | 0.3456 | No |
| 87 | TMEM119 |  |  | 1193 | 0.289 | 0.3462 | No |
| 88 | WASF2 |  |  | 1207 | 0.286 | 0.3462 | No |
| 89 | IL6ST |  |  | 1215 | 0.285 | 0.3479 | No |
| 90 | EVI2A |  |  | 1258 | 0.275 | 0.3392 | No |
| 91 | CPED1 |  |  | 1309 | 0.267 | 0.3279 | No |
| 92 | CEBPD |  |  | 1310 | 0.267 | 0.3315 | No |
| 93 | PPP1R18 |  |  | 1315 | 0.266 | 0.3339 | No |
| 94 | TMBIM1 |  |  | 1339 | 0.261 | 0.3306 | No |
| 95 | GLIPR1 |  |  | 1346 | 0.260 | 0.3323 | No |
| 96 | PSMB10 |  |  | 1375 | 0.256 | 0.3274 | No |
| 97 | ZFP36L2 |  |  | 1378 | 0.255 | 0.3302 | No |
| 98 | MSN |  |  | 1379 | 0.255 | 0.3336 | No |
| 99 | METTL7A |  |  | 1439 | -0.252 | 0.3195 | No |
| 100 | ARHGAP12 |  |  | 1497 | -0.257 | 0.3061 | No |
| 101 | KCTD12 |  |  | 1756 | -0.288 | 0.2336 | No |
| 102 | MIS18BP1 |  |  | 1764 | -0.289 | 0.2354 | No |
| 103 | RB1 |  |  | 1771 | -0.290 | 0.2375 | No |
| 104 | MED12L |  |  | 1773 | -0.290 | 0.2411 | No |
| 105 | A2M |  |  | 1808 | -0.295 | 0.2349 | No |
| 106 | FCGRT |  |  | 1871 | -0.301 | 0.2206 | No |
| 107 | TACC1 |  |  | 1916 | -0.307 | 0.2117 | No |
| 108 | SYNGR2 |  |  | 1939 | -0.312 | 0.2093 | No |
| 109 | CTSC |  |  | 1975 | -0.315 | 0.2032 | No |
| 110 | ADAM9 |  |  | 2063 | -0.328 | 0.1818 | No |
| 111 | NRIP1 |  |  | 2069 | -0.329 | 0.1847 | No |
| 112 | GATM |  |  | 2113 | -0.337 | 0.1765 | No |
| 113 | ARRB2 |  |  | 2235 | -0.360 | 0.1455 | No |
| 114 | PFN1 |  |  | 2305 | -0.373 | 0.1301 | No |
| 115 | BTG2 |  |  | 2318 | -0.375 | 0.1315 | No |
| 116 | PAG1 |  |  | 2347 | -0.383 | 0.1284 | No |
| 117 | UCP2 |  |  | 2498 | -0.413 | 0.0895 | No |
| 118 | SIRPA |  |  | 2562 | -0.431 | 0.0766 | No |
| 119 | IER2 |  |  | 2587 | -0.436 | 0.0753 | No |
| 120 | C12orf75 |  |  | 2607 | -0.441 | 0.0756 | No |
| 121 | GNAI2 |  |  | 2889 | -0.535 | -0.0005 | No |
| 122 | HEXA |  |  | 2894 | -0.537 | 0.0055 | No |
| 123 | GNB4 |  |  | 2943 | -0.568 | -0.0011 | No |
| 124 | SIPA1L2 |  |  | 2969 | -0.582 | -0.0007 | No |
| 125 | ITPR2 |  |  | 3002 | -0.603 | -0.0021 | No |
| 126 | APLP2 |  |  | 3034 | -0.621 | -0.0029 | No |
| 127 | CCND1 |  |  | 3072 | -0.655 | -0.0051 | No |
| 128 | SALL1 |  |  | 3190 | -0.768 | -0.0295 | No |
| 129 | GNG7 |  |  | 3220 | -0.807 | -0.0273 | No |
| 130 | GYPC |  |  | 3267 | -0.877 | -0.0292 | No |
| 131 | NINJ1 |  |  | 3372 | -1.108 | -0.0451 | No |
| 132 | HIST1H1C |  |  | 3427 | -1.335 | -0.0433 | No |
| 133 | PDGFB |  |  | 3428 | -1.343 | -0.0253 | No |
| 134 | PDPN |  |  | 3472 | -1.788 | -0.0141 | No |
| 135 | SORL1 |  |  | 3482 | -1.892 | 0.0086 | No |
Table: GSEA details [plain text format]

  

Fig 2: RICHARDS\_ZHONG\_NATURE\_2018\_MICROGLIA\_UPREG: Random ES distribution      
 Gene set null distribution of ES for **RICHARDS\_ZHONG\_NATURE\_2018\_MICROGLIA\_UPREG**
